# The Translational Relevance of Human Circulating Memory Cutaneous Lymphocyte-Associated Antigen Positive T Cells in Inflammatory Skin Disorders

**DOI:** 10.3389/fimmu.2021.652613

**Published:** 2021-03-23

**Authors:** Carmen de Jesús-Gil, Lídia Sans-de SanNicolàs, Irene García-Jiménez, Marta Ferran, Antonio Celada, Anca Chiriac, Ramon M. Pujol, Luis F. Santamaria-Babí

**Affiliations:** ^1^Translational Immunology, Department of Cellular Biology, Physiology, and Immunology, Faculty of Biology, Universitat de Barcelona, Parc Científic de Barcelona, Barcelona, Spain; ^2^Department of Dermatology, Hospital del Mar, Institut Hospital del Mar d'Investigacions Mèdiques (IMIM), Universitat Autònoma de Barcelona, Barcelona, Spain; ^3^Macrophage Biology, Department of Cellular Biology, Physiology, and Immunology, Faculty of Biology, Universitat de Barcelona, Barcelona, Spain; ^4^Department of Dermatophysiology, Apollonia University, Iasi, Romania

**Keywords:** skin-homing lymphocytes, cutaneous inflammation, CLA^+^ T cell, human, translational, immunodermatology

## Abstract

Circulating memory T cells are heterogeneous in their tissue tropism. The skin-seeking T cell subset expresses the cutaneous lymphocyte-associated antigen (CLA) on their surface. CLA^+^ memory T cells not only migrate from blood to skin but also recirculate between blood and skin. Studying CLA^+^ memory T cells in cutaneous diseases has allowed a better understanding of immune-inflammatory mechanisms that take place. The analysis of the phenotypical features of these cells, their antigen specificity, cytokine production profile, and changes in relationship to clinical status and therapies among other characteristics have led to the concept that they constitute peripheral cellular biomarkers in T cell-mediated cutaneous conditions. CLA^+^ memory T cells are of relevance in the pathogenesis of several cutaneous diseases, such as psoriasis (PSO), atopic dermatitis, vitiligo, and drug-induced allergic reactions, to name a few. The interaction of circulating CLA^+^ T cells with skin-resident cells has been investigated in different *ex vivo* coculture models made out of clinical samples. Interestingly, microbes that are present in the skin or related with human skin diseases are preferentially recognized by CLA^+^ T cells. Thus, the interaction of *Streptococcus pyogenes* with CLA^+^ T cells in PSO is providing novel concepts that help to understand disease immunopathogenesis. The goal of this review is to present latest results in the field of CLA^+^ T cells in T cell-mediated inflammatory skin diseases and their translational relevance for human immunodermatology.

## Introduction

**Figure 1:**
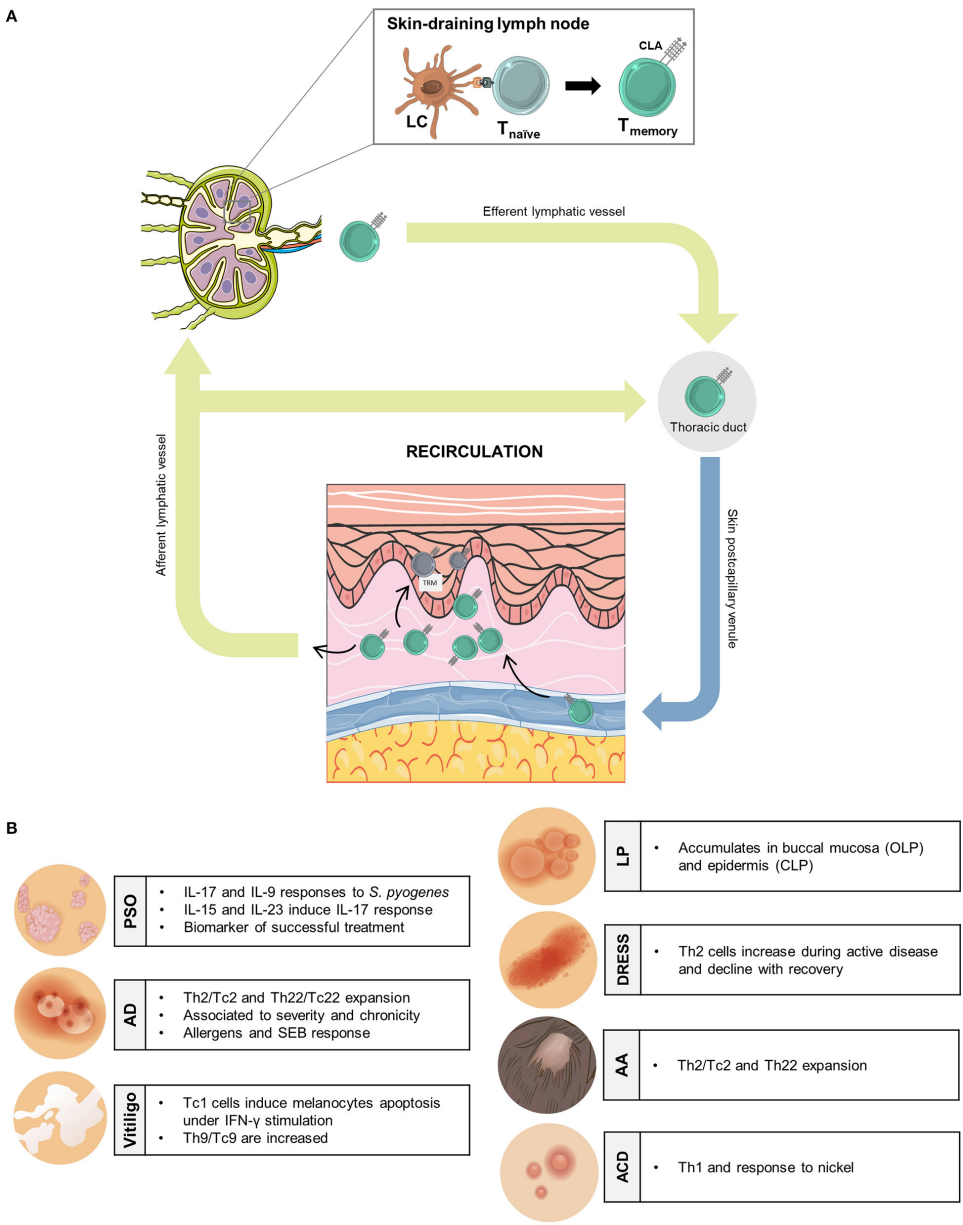
Translational relevance of human circulating memory CLA^+^ T cells in inflammatory skin disorders. **(A)** In skin-draining lymph nodes, antigen-specific Langerhans cells encounter naïve T cells generating memory T cells that express the skin-homing receptor CLA. CLA^+^ memory T cells enter the general circulation, through efferent lymphatic vessels, and will extravase to inflamed skin. Some CLA^+^ memory T cells leave cutaneous tissue through afferent lymphatic vessels, Recirculating back to the bloodstream, whereas, others are retained at the epidermal layer as resident memory T cells. **(B)** Principal antigen/allergen/autoantigen-induced effector functions of CLA^+^ memory T cells in human inflammatory cutaneous conditions. AA, alopecia areata; ACD, allergic contact dermatitis; AD, atopic dermatitis; CLA, cutaneous-associated lymphoid antigen; CLP, cutaneous lichen planus; DRESS, drug rash with eosinophilia and systemic symptoms; LC, Langerhans cells; OLP, oral lichen planus; PSO, psoriasis; T_RM_, T-resident memory. This figure was created using images from Smart Servier (https://creativecommons.org/licenses/by/3.0/), WikiHow (https://creativecommons.org/licenses/by-nc-sa/3.0/) and Freepik.es (@brgf).

The existence of a cutaneous immune system in humans was postulated almost 50 years ago (1) and recently reformulated (2). In order to understand the adaptive immune response regionally, for those human memory T cells that belong to the cutaneous immunity, a skin-specific cell marker would be of great help. The cutaneous lymphocyte-associated antigen (CLA) constitutes a relevant marker that identifies the subset of memory T lymphocytes functionally related to skin physiology. CLA was discovered in 1990 by serendipity as a cell surface carbohydrate preferentially expressed by T cells present in cutaneous inflamed tissues, but no other organs (3). Since then, a comprehensive number of scientific evidence described in humans support the role of CLA as a relevant marker to identify skin-associated memory T cells involved in T cell-mediated cutaneous inflammation, that provide translational information in numerous different human skin diseases. CLA is more than a mere cell surface carbohydrate (3), preferentially expressed on CD45RO^+^ T cells, which binds to endothelial E-selectin and mediates cell adhesion and transendothelial migration together with other molecules such as lymphocyte function-associated antigen-**1** (LFA-1), very late activation antigen-4 (VLA-4), and C–C chemokine receptor type 10 (CCR10) (4). CLA^+^ T lymphocytes are found in circulation and in inflamed and healthy skin but not infiltrating other non-cutaneous sites (3, 5). The fact that some CLA^+^ T cells are found in circulation deserves special attention due to the consequences of blocking LFA-1 in patients with atopic dermatitis (AD) and psoriasis (PSO) (6, 7). During treatment with efalizumab, patients present a circulating lymphocytosis of CLA^+^ T cells. If the treatment is interrupted, a flare in disease occurs. One explanation to this effect is that CLA^+^ T cells recirculate between blood and skin during cutaneous inflammation. Blocking their extravasation through LFA-1 leads to accumulation of those cells in blood, consequently when the blockade is released the cells enter abruptly into lesions and make flare. This mechanism of CLA^+^ T cell recirculation has important implications for the translational relevance of studying circulating CLA^+^ T cells in human inflammatory skin disorders (Figure 1A). CLA^+^ memory T cells participate in pathological mechanisms of inflammatory disorders by recognizing key triggers of disease and producing cytokines that affect cells of the skin. The phenotype of those cells can reflect clinical status of the patients. The goal of this review is to update this information on circulating CLA^+^ T cells in different human skin inflammatory diseases.

## Psoriasis

In PSO, CLA^+^ T cells are contributing to understand the pathological mechanisms from a translational point of view using *ex vivo* studies with clinical samples. Although PSO is considered to be triggered by cathelicidin (LL-37) (8) and interferon (IFN)-alpha (9, 10), the translation of this mechanism into patients is complex since anti-IFN-alpha (11), anti-IFN-gamma (12), and anti-IL-22 do not induce clinical improvement, to name a few clinically invalidated mechanisms. *Streptococcus pyogenes* (*S. pyogenes*) infection is the best characterized clinically relevant trigger of PSO in patients (13). The molecular mechanism that links *S. pyogenes* and IL-17 response is starting to be clarified in PSO (14). Only CLA^+^ memory T cells, but not CLA^−^, preferentially respond to *S. pyogenes* in an autologous coculture of T cells and cutaneous epidermal cells from patients with PSO (15). Besides, patients with PSO who are negative for antistreptolysin O (ASO) antibody present increases levels of immunoglobulin (Ig) A, but not IgG, to *S. pyogenes*, which are directly associated with CLA^+^ T–cell-dependent IL-17 response to *S. pyogenes in vitro* (16). These results suggest that increased exposure to *S. pyogenes*, as demonstrated by the presence of specific humoral immune response even in patients with ASO negative, upon recognition by CLA^+^ T cells can fuel pathogenic IL-17 production. Remarkably, similar association has been recently described for *Candida albicans*, which is a potent IL-23/Th17 inducer too (17). Some other new mechanisms in PSO relating CLA^+^ T cells and IL-17 have been recently revealed. The cytokine milieu present in psoriatic lesions can influence IL-17 response by CLA^+^ T cells. IL-15 and IL-23, both present in psoriatic lesions, have been shown to synergize with CLA^+^ T cells and autologous epidermal cells to produce IL-17A and IL-17F in PSO. This phenomenon occurs without the use of any exogenous stimulus, in a major histocompatibility complex (MHC)-dependent way, and independently of resident T cells (18), but it does not take place using CLA^−^ T cells or cells from healthy controls. This is an example of how psoriatic skin cytokine microenvironment interacts specifically with skin-related memory T cells generating the IL-17 response critical for PSO initiation and maintenance. IL-9 is another cytokine studied in the context of CLA^+^ T cells function in PSO in the *ex vivo* coculture model with autologous epidermal cells (19). *Streptococcus pyogenes* preferentially triggers IL-9 in CLA^+^ T cells together with other mediators such IL-17A. In contrast to the previous studies where IL-9 is not induced by natural stimulus, IL-9 is produced in a time-dependent way and not transiently. Interestingly, the neutralization of *S. pyogenes-*induced IL-9 by CLA^+^ T cells decreased IL-17A production by 50%.

Furthermore, the descriptive analysis of T cell subpopulations in patients with PSO revealed differential role for CD4^+^ and CD8^+^ T lymphocytes, whereas CLA expression is associated with skin recruitment of CD4^+^ central memory T cells (T_CM_), particularly those CCR4^+^ and CCR6^+^, suggesting a specific role for these cells in patrolling the skin compartment; CD8^+^ T cells are more likely to accumulate in psoriatic skin and stay as resident memory T cells (T_RM_) (20). Still, there is a need for psoriatic models, closely representing human disease, to be used as drug screening platforms. Recently, Shin et al. (21) developed psoriatic human 3D skin constructs (pHSCs) by incorporating T cells, over the classical approach based on the use of patient-derived keratinocytes or fibroblasts treated with PSO-related cytokines. As lymphocyte source, they tested *in vitro* polarized Th1/Th17 cells and CCR6^+^ CLA^+^ T cells from the patients with PSO, both of which showed psoriatic phenotype on epidermal cells along with disease-associated cytokine profile. Interestingly, when different psoriatic drugs were tested, those pHSCs with CCR6^+^ CLA^+^ T cells responded differently to the ones with *in vitro* polarized Th1/Th17 cells, highlighting the relevance of using patient-CLA^+^ T cells to better address and even anticipate specific therapeutical responses *in vitro*, moving one step forward to personalized medicine in PSO field.

The CLA^+^ lymphocytes have been extensively studied in the context of PSO disease, as last reviewed here (22). The importance of CLA^+^ T cells in PSO resides not only in their contribution to lesion formation but also in their role as peripheral biomarkers of successful treatment, as it has been confirmed by recent studies. For example, a significant reduction of circulating CLA^+^ T cell, as well as IL-6 and IL-22, has been associated with clinical efficacy after anti-tumor necrosis factor (TNF)-alpha treatment (23). Similarly, a reduction on the number of CLA^+^ Th17/Tc17 and Th22/Tc22 cells was observed after 6 weeks of phototherapy and balneotherapy, which positively correlated with the reduction on PSO area and severity index (PASI) score (24).

## Atopic Dermatitis

The CLA^+^ T cells are involved in initiation and perpetuation of AD (25), as they are functionally related to cutaneous inflammation (4, 26). Recent blood phenotyping studies on peripheral blood mononuclear cells from patients with AD and healthy controls (HC) have illustrated increased percentage of CLA^+^ memory T cells in moderate-to-severe patients with AD compared with age-matched HC, but a decrease with increasing age only in patients with AD (27, 28). The expansion of CLA^+^ T cells is accompanied by predominance of CLA^+^ Th2/Tc2 and Th22/Tc22 response in AD. Although CLA^+^ Th2 cell counts are similarly increased across all ages and are significantly higher than HC, CLA^+^ Th22 cell counts increase with age only in AD and its levels are also higher than that in HC (27–29). CLA^+^ Th22 levels correlate with severity parameters [scoring atopic dermatitis (SCORAD) and eczema area and severity index], pruritus, and IL-17-producing cells (29, 30). CLA^+^ IL-13^+^ T cells positively correlate with SCORAD, serum IgE levels, and IL-22 frequencies (27). Regarding Th1/Tc1 cell subsets, AD is characterized by decreased CLA^+^ Th1/Tc1 frequencies in conjunction with negative correlations between CLA^+^ IFN-gamma^+^ T cells and SCORAD, and CLA^+^ IL-13^+^ and IL-22^+^ populations. CLA^+^ Th1 frequencies increase with age both in AD and HC, but do not reach the HC levels, and they are associated with disease duration. All these data support an imbalanced CLA^+^ Th1:Th2 cell ratio that increases with age both in AD and HC, but remains decreased in AD (27–29).

The expression of the indicator of T cell mid activation, inducible costimulatory molecule (ICOS), has been reported to be enhanced both in CLA^+^ and CLA^−^ memory T cell subsets in patients with AD compared to HC and patients with PSO, predominantly in the skin-homing subset, and correlates with SCORAD. In contrast, the expression of human leukocyte antigen (HLA)-DR, the chronic T cell activation MHC class II antigen, is similar in infants regardless AD status, but increases with age reaching high levels in adult patients with AD vs. HC and patients with PSO, particularly among CLA^+^ T cell population, which correlates with SCORAD (28, 29, 31). Besides, frequencies of CLA^+^ regulatory T cells (T_regs_) are higher in AD compared with HC and patients with PSO, and they correlate with both ICOS and HLA-DR, and also with clinical parameters (SCORAD and IgE levels) (31). Intriguingly, OX40, another costimulatory molecule predominantly expressed on T cells and required for long-term memory responses, is mainly expressed by CLA^+^ memory T cells in both patients with AD and HC (32). A recent epigenetic report has extended the knowledge on the CLA^+^ T cells of male adult patients with severe AD and allergen-specific IgE sensitization (33). Peripheral blood mononuclear cells sorted into four different T cell populations (CD8^+^, CD4^+^, CD4^+^ CD45RA^+^, and CD4^+^ CLA^+^) have revealed differentially DNA methylations in 40 protein-coding genes in the CD4^+^ CLA^+^ subset in patients with AD vs. HC. Among them, IL-13 gene promoter shows decreased methylation levels, which negatively correlate with IL13 mRNA expression levels in this subset.

## Vitiligo

Melanocyte-specific circulating memory CD8^+^ CLA^+^ T cells induce melanocyte apoptosis, together with other mechanisms, such as cell detachment triggered by E-cadherin disruption (34), contribute to melanocyte loss and the development of depigmented skin lesions in vitiligo. Several blood endotyping studies have depicted that patients with vitiligo have low frequencies of circulating CD4^+^/CD8^+^ CLA^+^ T_EM_/T_CM_ cells compared to patients with PSO, being similar to HC, supporting that CLA^+^ T cells migrate to the skin (35, 36). Within T cell subsets, patients with vitiligo have the highest frequency of CD4^+^/CD8^+^ CLA^+^ T cells producing IFN-gamma compared with patients with AD, PSO, and alopecia areata (AA), and HC (35), supporting that vitiligo is caused by a type 1 T cell response (37, 38). Consistent with the literature describing CD8^+^ T cell role in melanocytes death under IFN-gamma stimulation, CD8^+^ CLA^+^ T cell population producing IFN-gamma predominates over CD4^+^ CLA^+^ T cell subset in patients with vitiligo (35). Interestingly, the same study showed the highest frequency of CD4^+^/CD8^+^ CLA^+^ T cells producing IL-9 in patients with vitiligo in comparison with patients with AD, PSO, and AA and HC, pointing out for the first time a possible role for IL-9 on the physiopathology of vitiligo. Furthermore, Th17 subset, either CLA^+^ or CLA^−^, was also increased in vitiligo compared to patients with HC, AD and, surprisingly, with PSO, a disease associated to be driven by IL-17 activation. Regarding CLA^+^ Th22 subset, it was also augmented in patients with vitiligo compared to patients with PSO and HC (35). In accordance to this fact, IL-22 has been reported to participate in the pathogenesis of vitiligo, as it promotes IL-1β secretion from keratinocytes what cause the suppression of melanogenesis and melanocyte migration as well as the induction of melanocyte apoptosis (39). The active participation of autoreactive CD8^+^ T cells in vitiligo indicates that immune tolerance has been disrupted. Several studies have shown that patients with vitiligo have a reduced amount of infiltrating T_regs_ in non-lesional, perilesional, and lesional skin (40, 41). Patients with vitiligo also have fewer amounts of total circulating T_regs_ compared to patients with AD and PSO, the difference being not that obvious in the CLA^+^ T_regs_ subset (35).

## Alopecia Areata

The CLA relevance in AA in humans was first revealed by Yano et al. (42), describing higher positivity in peripheral blood mononuclear cells, CD4^+^ and CD8^+^ lymphocytes from AA compared with HC, and that CLA positivity negatively correlated with clinical improvement. Later, phenotyping research on peripheral blood mononuclear cells described that CLA^+/−^ Th2-cell frequencies are similar between patients with AA and AD, both higher than HC, and they correlate with AA severity; however, skin-homing Tc2 and Th22 are significantly higher in AA vs. HC (43). Also, the positive correlations have been reported not only between CLA^+^ Th2/Tc2 and Th22/Tc22 but also between CLA^+^ Th17/Tc17 and Th22/Tc22, particularly in the CD8^+^ subset. In addition to this, skin-homing CD4^+^/CD8^+^ T_CM_-cell counts are higher in patients with AA, compared with HC and patients with AD, unlike CD4^+^/CD8^+^ T_EM_ frequencies, and CD4^+^, but not CD8^+^, subset shows HLA-DR activation in T_CM_ cells. Regarding T_regs_ cells, diminished frequencies of total and CLA^+^ T_regs_ have been described in patients with AA compared to HC and patients with AD, which correlate with skin-homing Th9, Th2/Tc2, Tc17, and Tc22 counts.

## Other Inflammatory Skin Diseases

In lichen planus, there is an accumulation of CLA^+^ T cells in the epithelium of the buccal mucosa in oral lichen planus (OLP), and in the epidermis of skin biopsies in cutaneous lichen planus (CLP) (44). Increased E-selectin expression in lesional biopsies from OLP over perilesional tissue, together with a significantly higher proportion of CD8^+^ CLA^+^ T cells were observed by the immunohistochemical analysis. It has been shown cutaneous T cell-attracting chemokine (CTACK, also CCL27) secretion by oral keratinocytes, which increased in the presence of IFN-gamma and actively attracted CLA^+^ memory T cells to the oral epithelium. Although both normal oral mucosa and lesions from chronic OLP showed low levels of CTACK expression, it may still play a role in early recruitment of T cells and immunopathogenesis of OLP (45).

Although the role of CLA^+^ T cells in non-immediate drug-induced cutaneous reactions was first reported two decades ago (46), there is still a lot to learn about them in drug allergy. Recently, it has been shown that in drug rash with eosinophilia and systemic symptoms (DRESS) (47), IL-4 and IL-13 producing CD4^+^ T cells are increased during active disease and decline with recovery, pointing at the relevance of CLA^+^ Th2 cells in the pathogenesis of DRESS. In β-lactam hypersensitivity, a new T cell subset has been proposed, in which blood- and skin-derived clones specific for piperacillin expressed high levels of skin-homing chemokine receptors and migrated in the presence of the ligands CCL27 (48).

## Discussion

The involvement of CLA^+^ T cells in different inflammatory skin disorders, with diverse pathological immunological mechanisms, makes them interesting for human dermatology (Figure 1B). Also, circulating memory CLA^+^ T cells specific for disease relevant antigens/allergens have reported, for example, *S. pyogenes* in PSO (22) or house dust mite and *Staphylococcus aureus* enterotoxin B in AD (4). Despite the functional relevance of memory CLA^+^ T cells has been better described in PSO and AD (4, 22, 49); their potential role in other T cell-mediated skin conditions is still to be fully investigated. Various approaches that involve minimal manipulation of those cells are providing immunological information that relates to clinic in a translational way. Although animal models and complex *in vitro* models are providing important information to understand human inflammatory skin disorders, there is a need to use clinical human samples to gather genetic background and real diseased skin cells of patients (50). For example, animal models cannot reflect the immune response present in patients after several years of chronic cutaneous inflammation and numerous flares. Different aspects can influence the immune response in inflammatory skin diseases such as genetic background, disease endotype/patient heterogeneity, local antigen presentation by human epidermal cells, impact of environmental triggers of disease, or neurogenic inflammation. This integrated view is the approach that CLA^+^ T cells exploration follows.

Nonetheless, the interplay between circulating and tissue-resident CLA^+^ T cells should be further addressed. Particularly since the latter have been described to persist in resolved skin after treatment and to be involved in the recurrence of cutaneous lesions in PSO, vitiligo, or fixed drug eruptions (51).

We are clearly still at the top of the iceberg unraveling the information that those lymphocytes can provide for human inflammatory cutaneous conditions. For all these translational capacities, circulating memory CLA^+^ T cells can be proposed as peripheral cellular biomarkers in human inflammatory skin disorders.

## Author Contributions

CJ-G and LS: equal contribution writing and experimental. LS-B: principal investigator, director, founding, and corresponding author. RP: clinical, translational work, and founding. ACh: clinical and translational work. ACe: immunology expertise. MF: clinical and translational work. IG-J: contribution writing. All authors contributed to the article and approved the submitted version.

## Conflict of Interest

The authors declare that the research was conducted in the absence of any commercial or financial relationships that could be construed as a potential conflict of interest.
